# Totum-070, a Polyphenol-Rich Plant Extract, Prevents Hypercholesterolemia in High-Fat Diet-Fed Hamsters by Inhibiting Intestinal Cholesterol Absorption

**DOI:** 10.3390/nu15245056

**Published:** 2023-12-09

**Authors:** Cédric Langhi, Marie Vallier, Yolanda F. Otero, Maheva Maura, Florian Le Joubioux, Hugo Groult, Oussama Achour, Ratna Budhi Pebriana, Martin Giera, Bruno Guigas, Thierry Maugard, Benoit Chassaing, Sébastien Peltier, Jean-Marie Bard, Pascal Sirvent

**Affiliations:** 1 R&D Riom Center, Valbiotis, 20-22 rue Henri et Gilberte Goudier, 63200 Riom, France; 2 R&D Center, Valbiotis, 23 Avenue Albert Einstein, 17000 La Rochelle, France; 3 Equipe BCBS (Biotechnologies et Chimie des Bioressources pour la Santé), UMR (Unité Mixte de Recherche) CNRS (Centre National de la Recherche Scientifique) 7266 LIENSs (LIttoral ENvironnement Et Sociétés), La Rochelle Université, 17042 La Rochelle, France; 4 BioAqtiv, Equipe BCBS (Biotechnologies et Chimie des Bioressources pour la Santé), LIENSs (LIttoral ENvironnement Et Sociétés), UMR (Unité Mixte de Recherche) 7266 CNRS (Centre National de la Recherche Scientifique), La Rochelle Université, 17042 La Rochelle, France; 5 Center for Proteomics and Metabolomics, Leiden University Medical Center, Albi-nusdreef 2, 2333 ZA Leiden, The Netherlands; 6 Department of Parasitology, Leiden University Medical Center, Albinusdreef 2, 2333 ZA Leiden, The Netherlands; 7 Team “Mucosal Microbiota in Chronic Inflammatory Diseases”, Institut Cochin, INSERM (Institut National de la Santé et de la Recherche Médicale) U1016, CNRS UMR 8104, Université Paris Cité, 75014 Paris, France; 8 R&D Périgny Center, Valbiotis, 12F rue Paul Vatine, 17180 Périgny, France; 9 Laboratoire de Biochimie Générale et Appliquée, UFR (Unité de Formation et de Recherche) de Pharmacie, ISOMer-UE 2160, IUML-Institut Universitaire Mer et Littoral-FR3473 CNRS, Université de Nantes, 44035 Nantes, France

**Keywords:** triglycerides, lipoproteins, liver, dyslipidemias, enterocytes, polyphenols, nutraceutical

## Abstract

Atherosclerotic cardiovascular disease is the leading cause of mortality worldwide, and hypercholesterolemia is a central risk factor for atherosclerosis. This study evaluated the effects of Totum-070, a plant-based polyphenol-rich supplement, in hamsters with high-fat diet (HFD)-induced dyslipidemia. The molecular mechanisms of action were explored using human Caco2 enterocytes. Totum-070 supplementation reduced the total cholesterol (−41%), non-HDL cholesterol (−47%), and triglycerides (−46%) in a dose-dependent manner, compared with HFD. HFD-induced hepatic steatosis was also significantly decreased by Totum-070, an effect associated with the reduction in various lipid and inflammatory gene expression. Upon challenging with olive oil gavage, the post-prandial triglyceride levels were strongly reduced. The sterol excretion in the feces was increased in the HFD-Totum-070 groups compared with the HFD group and associated with reduction of intestinal cholesterol absorption. These effects were confirmed in the Caco2 cells, where incubation with Totum-070 inhibited cholesterol uptake and apolipoprotein B secretion. Furthermore, a microbiota composition analysis revealed a strong effect of Totum-070 on the alpha and beta diversity of bacterial species and a significant decrease in the Firmicutes to Bacteroidetes ratio. Altogether, our findings indicate that Totum-070 lowers hypercholesterolemia by reducing intestinal cholesterol absorption, suggesting that its use as dietary supplement may be explored as a new preventive strategy for cardiovascular diseases.

## 1. Introduction

Atherosclerotic cardiovascular disease (ASCVD) is the main cause of mortality worldwide [1,2]. It is well-established that an increased plasma low-density lipoprotein cholesterol (LDL-C) level is the primary risk factor for the development of coronary heart disease and atherosclerosis [3,4]. Therefore, lifestyle modifications, particularly a healthy diet, are important steps in ASCVD primary prevention. However, lifestyle changes are not easily achieved and maintained. In people with moderate cholesterol levels, dietary supplements may contribute to improving the health status in addition to a balanced diet. In recent years, many animal studies and clinical trials have shown the beneficial effects of polyphenolic compounds as cholesterol-lowering agents [5,6,7,8]. Many plants have been explored to reduce blood cholesterol, such as artichokes [9,10], olive leaves [11,12], and goji berries [13]. The hypolipidemic effect of artichoke leaf extracts has been well described in various animal models, such as high-fat-fed [14,15] and alloxan-induced diabetic rats receiving a daily gavage of artichoke leaf extract [16]. Furthermore, Qiang et al. demonstrated that Golden Syrian hamsters fed a cholesterol-enriched diet supplemented with artichoke leaf extract prevented hypercholesterolemia [17]. The effect of olive leaf extract on lipid metabolism has been described in HFD-fed and ovariectomized rats supplemented with olive leaf extract [18,19]. Wang et al. also showed that New Zealand rabbits fed an HFD for 6 weeks with olive leaf extract exhibited a reduction in hyperlipidemia [20]. The hypolipidemic effect of Goji fruit extract was demonstrated in rats fed an HFD for 8 weeks [21]. This effect was reproducible in other studies with rats fed a cholesterol-enriched diet for 45 days [22] or for 60 days [23]. Luo et al. demonstrated in alloxan-induced diabetic rabbits that 10 days of oral administration of Goji fruit extract reduced hypercholesterolemia [24]. Finally, the hypocholesterolemic effect of black pepper has been reported in various animal models [25,26]. 

Totum-070 is a polyphenol-rich formulation based on five plant extracts (olive leaves (*Olea europaea* L.), artichoke leaves (*Cynara scolymus* L.), chrysanthellum, aerial part, (*Chrysanthellum indicum* subsp. *afroamericanum* B.L. *Turner*), goji fruits (*Lycium barbarum*), and black pepper fruits (*Piper nigrum* L.)) to lower cholesterol in individuals with mild to moderate hypercholesterolemia (French priority patent number: FR1559965). The aim of this study was to evaluate the effects of a 12-week supplementation with Totum-070 on hypercholesterolemia in hamsters fed a high-fat diet (HFD) and to decipher the mechanisms of Totum-070’s effect on lipoprotein metabolism.

## 2. Materials and Methods

### 2.1. Totum-070 (Lipidrive^®^) Characterization

The batch used in this study was chemically characterized using the Folin–Ciocalteu method (total polyphenol estimation), the Dubois method (total sugar estimation), the phospho–vanillin method (total lipid estimation) [27,28], the O-phthalaldehyde method (total protein estimation) [29], and HPLC-UV/visible/MS (Agilent Technologies Infinity series 1200 and 1260, Santa Clara, CA, USA) with a C18 column (250 × 4.6 mm, 5 μm, Phenomenex, Torrance, CA, USA) and a HILIC Silica column (150 × 4.6 mm, 5 μm, Waters, Etten-Leur, The Netherlands) (quantification of potential compounds of interest). The results of this analysis are presented in Table 1. Totum-070 is available from Valbiotis (Perigny, France) under the brand name Lipidrive^®^.

### 2.2. Animals

All the animal procedures were approved by the local ethics committee (C2E2A, Auvergne, France, under the number 18588-2019012216136952V5 dated 2 October 2020) and comply with the ARRIVE guidelines. Male golden Syrian hamsters were purchased from Janvier Labs (Le Genest-Saint-Isle, France). All the hamsters were housed at the animal facility center (Valbiotis R&D Center, Riom, France) at 22 °C under a standard 12 h light–12 h dark cycle, 2 or 3 hamsters per cage. Upon arrival, the hamsters were fed a normal diet (ND, D18102403, Research Diets, New Brunswick, NJ, USA) for 2 weeks of acclimatization until the age of 8 weeks. Thereafter (i.e., study start), the animals were randomly assigned to five groups with similar mean body weight and fat mass: (i) ND (*n* = 15); (ii) HFD (45 kcal% fat, mainly hydrogenated coconut oil, 17 kcal% fructose, 1.4 gm% cholesterol; D99122211, Research Diets, New Brunswick, NJ, USA) (*n* = 16); (iii) HFD supplemented with 3% (*wt*/*wt*) of Totum-070 (HFD-T070 3%; *n* = 16); (iv) HFD supplemented with 4.25% (*wt*/*wt*) of Totum-070 (HFD-T070 4.25%; *n* = 16); and (v) HFD supplemented with 5% (*wt*/*wt*) of Totum-070 (HFD-T070 5%; *n* = 16). Food and water were supplied ad libitum. Supplementation was for 12 weeks. The hamsters were weighed every week. Fresh food was distributed every 2 to 3 days and food intake was recorded. The ND and HFD energy densities were 3.85 kcal∙g^−1^ and 4.5 kcal∙g^−1^, respectively. The lipid parameters were monitored in the blood before the study’s start (week 0), after 6 weeks of supplementation (week 6), and at the study’s end (week 12). The hamsters were fasted for 6 h, and blood was collected from the gingival vein. Then, the blood was incubated at room temperature for 30 min and centrifuged at 2000× *g* for 10 min. The serum was harvested and stored at −80 °C until analysis. The body composition was assessed with an echo MRI system (Zynsser Analytic) at week 0, week 6, and week 12. Feces were collected from each cage during 3 full days (from day 75 to day 78). At the study’s end, the animals were fasted for 6 h, and then anaesthetized with isoflurane. Blood was collected by cardiac puncture, and then the hamsters were sacrificed by cervical dislocation.

### 2.3. Serum Lipid Parameters

High-density lipoprotein (HDL)-enriched serum fractions were isolated by incubating the serum samples with the LDL/VLDL precipitation buffer (Abcam, Boston, MA, USA). The cholesterol levels in the serum (total cholesterol) and in the purified HDL fraction (HDL-C) were quantified enzymatically using the CHOD-PAP colorimetric assay kit (Biolabo SAS, Maizy, France). The non-HDL-C fraction of each sample was calculated by subtracting the HDL-C concentration from the total cholesterol concentration. The serum triglyceride concentration was measured with a colorimetric assay kit (Cayman chemical, Ann Arbor, MI, USA).

### 2.4. Liver Triglycerides

The liver triglyceride level was determined with the triglyceride enzymatic assay kit by Cayman Chemical. The hamster livers (100 mg) were homogenized in 1 mL of a solution containing 5% NP40 in water, followed by centrifugation (10,000× *g* at 4 °C for 10 min) to recover the supernatant. The triglyceride concentration was normalized to the liver sample weight and expressed as mg/mg liver weight.

### 2.5. Histology

At the study’s end, the liver tissue was fixed in paraformaldehyde at 4 °C for 48 h and kept in 70% ethanol for 2 to 3 h before automated tissue processing. The samples were embedded in paraffin, and 4 μm sections were cut with a microtome. The sections were dewaxed and rehydrated in successive xylene and ethanol baths. Then, they were stained in Gill II hematoxylin for 4 min, and after the removal of excess stain in an alcohol acid bath, they were transferred to a 0.25% eosin Y bath for 45 s. Finally, the sections were dehydrated with ethanol and xylene, and slides were mounted with Eukitt medium. Other parts of the livers were frozen in OCT medium, and 10 μm tissue sections were cut with a cryostat and fixed in cold 10% formalin for 5 min. The slides were stained with Oil Red O at 60 °C in an oven for 8 min. The Oil Red O areas were quantified using ImageJ software (version: v1.54g). Stained areas of at least 10 μm^2^ and with a circularity between 0.1 and 1 were selected for quantifying the Oil Red O surface as a percentage of the total surface. At least three images per liver were analyzed.

### 2.6. Fecal Neutral Sterols 

The fecal neutral sterols were quantified in the feces collected from 5 HFD cages and 5 HFD–T070 5% cages (*n* = 2 or 3 hamsters per cage). The feces were dried at 60 °C for 72 h and weighted. The sterols were analyzed as previously published with minor modifications in the selected ion monitoring mode [30]. Briefly, 0.5 mg of fecal homogenate in 90% *v*/*v* isopropanol was hydrolyzed using 1 M NaOH in 80% *v*/*v* of ethanol. Then, the sterols were extracted twice with methyl tert-butyl ether and derivatized with a mixture of *N*-methyl-*N*-(trimethylsilyl)trifluoroacetamide, trimethylsilyl chloride, and *N*-trimethylsilyl-imidazole. Cholestane, cholesterol-d7, desmosterol-d6, and 25-hydroxy cholesterol-d6 were used as internal standards, and the sterols were quantified against external calibration lines. The sterol concentrations were expressed as µg/mg of dry feces. The calculated fecal neutral sterol excretion was expressed per cage and as µg/day.

### 2.7. Serum Lipopolysaccharide (LPS) Load Quantification

The LPS concentration was determined in the serum obtained from cardiac puncture at the study’s end, as described elsewhere [31]. Hek-blue mTLR4 cells (from InVivogen, San Diego, CA, USA) were plated in 96-well plates (25,000 cells/well) in 180 µL of detection medium/well. After 24 h, 20 µL of LPS standard or diluted serum sample was added to 180 µL of detection medium to each well in duplicate. After 24 h of incubation at 37 °C with 5% CO_2_, the absorbance was measured at 630 nm using a Spark microplate reader (Tecan, Männedorf, Switzerland).

### 2.8. Intestinal Cholesterol Absorption Test

The intestinal cholesterol absorption test was performed according to the dual-isotope plasma ratio method described by Turley et al. with modifications [32,33]. Briefly, at the study’s end, the non-fasted hamsters were anesthetized with isoflurane. Then, 2.5 μCi of [1,2-^3^H(N)]-cholesterol (Perkin Elmer, Every, France), completely dissolved in 200 μL of intralipid (I141, Sigma-Aldrich, Saint-Louis, MO, USA), was injected directly in the gingival vein. Immediately afterwards, 1 μCi [4-^14^C]-cholesterol (Perkin Elmer, Every, France) dissolved in 600 µL olive oil was administered by gavage. The hamsters were returned to their cage, where they received their assigned diet for 72 h. Then, the hamsters were anesthetized with isoflurane and were bled from the heart to collect blood, which was then incubated at room temperature for 30 min and centrifuged at 2000× *g* for 10 min. To determine the proportion of [^14^C]- and [^3^H]-cholesterol still in circulation after 72 h, 300 µL of each serum sample and the original mixtures were added to 3 mL Ultima Gold liquid scintillation cocktails (Perkin Elmer, Every, France). The vials were shaken vigorously, and the radioactivity was counted using a TRI-CARB 4910TR Liquid Scintillation Counter (Perkin Elmer, Every, France).

### 2.9. Oral Fat Tolerance Test 

This test was performed at week 11. After overnight fasting, the hamsters received 300 µL of olive oil by gavage, and the triglyceride concentration in the serum was quantified before (0), 150, 330, 510, and 720 min after gavage. 

### 2.10. Hepatic Triglyceride Production Test 

After 12 weeks of supplementation, the triglyceride secretion rate in the blood was quantified using Poloxamer 407. This nonionic detergent inhibits the tissue lipoprotein lipase and, thus, the removal of triglycerides from the circulation [34]. A 100 g/L Poloxamer 407 solution in an injection-grade saline solution was injected intraperitoneally in overnight-fasted hamsters (1000 mg/kg body weight). Blood samples were collected immediately (0) and at 45, 90, and 120 min after injection to quantify the triglyceride concentration. The liver triglyceride secretion rate was calculated by multiplying the slope of the triglyceride concentration increase over time by the intravascular distribution volume (estimated at 3.8 mL/100 g body weight) [35,36].

### 2.11. Lipoprotein Lipase Activity Assay

The post-heparin lipoprotein lipase activity was measured using a previously described method [37,38]. After 12 weeks, the hamsters were fasted for 4 h and then anesthetized with isoflurane. Then, 100 µL (40 U) of sodium heparin (Sanofi, Paris, France) was injected into the gingival vein. After 5 min (to allow lipoprotein lipase release in the plasma), the animals were sacrificed, then blood was collected by cardiac puncture and separated by centrifugation at 2000× *g* at 4 °C for 10 min. The total lipase activity was determined using the LPL assay kit (Abcam, Boston, MA, USA). The hepatic lipase activity was then determined by incubating the reaction mixture in the presence of 1 M NaCl, which specifically blocks LPL activity. The lipoprotein lipase activity was calculated as the difference between the total lipase activity and hepatic lipase activity [38,39].

### 2.12. Quantitative Reverse Transcription-PCR (RT-qPCR)

The total mRNA was extracted from snap-frozen tissues using TRIzol^®^ (Invitrogen (Waltham, MA, USA), Life Technologies (Carlsbad, CA, USA)). cDNA was synthesized from 2 μg RNA with a high-capacity cDNA transcription kit (Applied Biosystems (Waltham, MA, USA), Life Technologies). PCR amplification was carried out using the CFX system (Bio-Rad, Marnes-la-Coquette, France) with SybrGreen primers (Eurofins, Luxembourg). The ΔΔCt method was used to quantify the mRNA levels. Gene expression was normalized to the housekeeping gene *Polr2a*. The data are presented using the Rq, normalized to the control group, with the following equation:

Rq = 2^−ΔΔCt^ [ΔCt = Ct (target) − Ct (*Polr2a*); ΔΔCt = ΔCt (sample) − ΔCt (control)].

The primer list is in Table S4. 

### 2.13. Protein Extraction and Western Blot Analysis

The liver samples were homogenized in 20 µL/mg RIPA buffer (50 mM Tris-HCl, 150 mM NaCl, 1% NP40, 0.5% sodium deoxycholate, 0.1% SDS) on ice using a glass potter. Just before use, protease (P8340, Sigma, Saint-Louis, MO, USA) and phosphatase (88667, Thermo Fisher Scientific, Waltham, MA, USA) inhibitors were added to the buffer. The homogenized samples were centrifuged at 14,000× *g* at 4 °C for 10 min, and the supernatant was collected. The membrane proteins were extracted using the Mem-PER Plus Membrane Protein Extraction Kit (Thermo Fisher Scientific, Waltham, MA, USA), following the manufacturer’s protocol. The protein content was determined using a commercial Lowry-based DC protein assay (Bio-Rad, Marnes-la-Coquette, France). All the samples were adjusted to a standard concentration (15 µg) and then diluted with 2× Laemmli buffer. Western blotting was performed as described by Chavanelle et al. [40] with the following specificities: the membranes were incubated at 4 °C with primary antibodies against an LDL receptor (Biovision, Milpitas, CA, USA), MTTP (Abcam, Boston, MA, USA), ACAT2 (Cell signaling, Saint-Cyr-L’Ecole, France), and NPC1L1 (Cell signaling, Saint-Cyr-L’Ecole, France), all at 1:1000. After overnight incubation, the membranes were washed with Tris-buffered saline/0..% Tween-20 and incubated with anti-rabbit horseradish peroxidase-conjugated secondary antibodies (1:2000) at room temperature for 1 h. Then, the membranes were washed three times in Tris-buffered saline/0.5%Tween-20 before the addition of an enhanced chemiluminescent solution (Clarity Western ECL; Bio-Rad) for 1 min. Band images were acquired with the Bio-Rad ChemiDoc system, and the band density was determined using Image Lab V6.0 (Bio-Rad). Stain-free blot images were used as the total protein loading control for data normalization [41]. 

### 2.14. Cell Cultures

Human colon colorectal adenocarcinoma Caco-2 cells (Sigma, Saint-Louis, MO, USA) were maintained in DMEM 4.5 g/L glucose supplemented with 2 mM glutamine, 1% nonessential amino acids, 20% fetal bovine serum, and 1% penicillin/streptomycin. For the experiments, Caco-2 cells were seeded on 1.0 µm transparent polyethylene terephthalate membrane inserts and allowed to differentiate for 21 days. Radioactive micelles were prepared freshly according to [42]. Briefly, lipids were added to the cells in the apical pole in the DMEM/serum-free medium as a complex lipid emulsion containing 0.4 mM oleic acid, 0.2 mM L-α-lysophosphatidylcholine, 0.05 mM cholesterol, 0.2 mM 2-oleoylglycerol, and 2 mM taurocholic acid and supplemented with [1,2-^3^H(N)]-cholesterol (2 µCi/mL). For the [^3^H]-cholesterol uptake assay, Caco-2 cells were incubated with radioactive micelles and with/without Totum-070 for 1 h. Then, the cell layers were washed, and the cell lysates were collected for protein determination. The cell lysates were mixed directly in the liquid scintillators (Ultima Gold, Perkin Elmer, Every, France). The radioactivity was counted with a TRI-CARB 4910TR Liquid Scintillation Counter and normalized to the cell protein content. In some conditions, ezetimibe (Sigma, Saint-Louis, MO, USA) was added directly in the apical medium as a positive control of [^3^H]-cholesterol uptake inhibition. For the [^3^H]-cholesterol secretion assay, the Caco-2 cells were first loaded with radioactive micelles in the upper compartment for 24 h. Then, the cells were washed, and the apical medium was changed to DMEM containing Totum-070 at the indicated concentration. After overnight incubation, the basolateral medium was collected and the lipoproteins secreted in the basolateral medium were separated from the free tracers with a PD-10 Sephadex-G25 desalting column (Sigma, Saint-Louis, MO, USA) [43]. The purified fraction was suspended in a liquid scintillator (Ultima Gold, Perkin Elmer, Every, France), and the radioactivity was counted with a TRI-CARB 4910TR Liquid Scintillation Counter. The radioactivity in each vial was normalized to the cell protein content. For the apolipoprotein B (ApoB) secretion measurement, a protocol similar to the one for cholesterol secretion quantification was used, but without radiolabeled cholesterol during the cell loading with micelles. The ApoB secretion into the basolateral medium after overnight incubation was quantified with the Human Apolipoprotein B ELISA Kit (Abcam, Boston, MA, USA). MTTP and ACAT2 were detected by Western blotting using cells in the same culture condition described above. For NPC1L1 detection by Western blotting in whole-cell and in the cell membrane lysates, a protocol similar to the one for the cholesterol uptake assay was used but with non-radioactive micelles.

### 2.15. RNA Sequencing Analysis

The livers were collected from the euthanized hamsters after 12 weeks of supplementation and immediately frozen in liquid nitrogen. The RNA purity and concentration were determined using a NanoDrop ND-2000 Spectrophotometer (Thermo Fisher Scientific) and a Qubit fluorometer (Thermo Scientific), respectively. The RNA integrity was assessed on an RNA 6000 NanoChip using a 2100 Bioanalyzer (Agilent Technologies). The rRNA was depleted from 200 ng of the total RNA using a NEBNext rRNA Depletion Kit (Human/Mouse/Rat) (New England BioLabs, Ipswich, MA, USA) following the manufacturer’ protocol. Fragmented double-strand cDNA was synthesized using a NEBNext Ultra II Directional RNA Library Prep Kit (New England Biolabs) according to the manufacturer’s instructions. The libraries were amplified for 9 cycles and purified using Ampure XP beads (Beckman Coulter, Brea, CA, USA), and their concentration was measured using a Qubit 2.0 Fluorometer and a Qubit dsDNA HS Assay Kit (Thermo Fisher Scientific). Then, the library quality was confirmed by a size analysis on a Bioanalyzer 2100 instrument and a DNA 1000 Assay reagent kit (Agilent Technologies). These data were used for pooling libraries at equimolar concentrations to normalize each library. Single-end, 75-cycle sequencing was performed on a NextSeq 500 Sequencing System using NextSeq 500/550 High Output v2 kit (75 cycles) (Illumina, San Diego, CA, USA). FASTQ files were generated using bcl2fastq (Illumina). The reads were trimmed with Trimmomatic (version 0.36) to filter poor quality reads and to cut poor quality bases and adapters. The filtered reads were mapped to the reference genome (*Mesocricetus auratus*, assembly MesAur1) using STAR (version 2.7.10). The STAR software was also used to produce the count matrix to assign reads to features using transcriptome annotations from the GTF file. The quality control statistics were summarized using MultiQC (version: v1.14). The descriptive analysis was performed with the DESeq2 R package. The differential analysis was performed with the edgeR R package. TMM normalization was applied. The glmQLFit and glmQLFTest functions were used to identify differentially expressed genes in the three groups (ND, HFD, and HFD-T070 5%). The genes of interest were selected based on the significance (Benjamini–Hochberg correction, corrected *p*-value < 0.05) and effect size (fold-change) for each comparison. The gene lists were annotated with the DAVID database (database for annotation, visualization, and integrated discovery, https://david.ncifcrf.gov/home.jsp, accessed on 1 February 2022) and *Cricetulus griseus* ortholog gene identifiers.

### 2.16. Microscale Thermophoresis (MST) Binding Affinity Assay

The binding affinity of Totum-070 for the *N*-terminal domain (22–284 amino acids) of the NPC1L1 protein (NPC1L1-NTD) was assessed using MST. Recombinant human NPC1L1-NTD (MBS7102155, MyBiosource, San Diego, CA, USA) was labeled with the fluorescent RED-NHS dye using a RED-NHS protein labeling kit (NanoTemper Technologies, Munich, Germany) according to the manufacturer’s instructions. Briefly, the dye and NPC1L1-NTD were incubated, and the tagged protein was purified. The resultant tagged solution was then diluted in a binding buffer (50 mM Tris-HCl pH 7.5, 150 mM NaCl, 1 mM DTT, and 0.1% Fos-choline 13) to achieve a final NPC1L1-NTD concentration of 20 nM in the capillaries. Standard capillaries for red fluorescence emission recording were filled with a 1:1 (*v*/*v*) mixture of tagged NPC1L1-NTD and diluted ligand. The fluorescence was measured using a NanoTemper Monolith NT.115 instrument (NanoTemper Technologies, Munich, Germany) at 37 °C, with a fluorescence excitation power of 90% and the MST power set to high. The data were analyzed with NanoTemper analysis software(version: v2.3) (NanoTemper Technologies, Munich, Germany).

### 2.17. Microbiota Analysis

The cecum content was harvested from the euthanized hamsters after 12 weeks of supplementation and immediately frozen in liquid nitrogen. DNA was extracted from 50–100 mg of the sample using a FastDNA^TM^ Spin Kit for feces (MP Biomedicals, Illkirch, France) and FastPrep-24 5G (MP Biomedicals, Illkirch, France) following the manufacturer’s instructions. The DNA was eluted in 50 µL of elution buffer, pre-heated at 55 °C, and normalized to 20 ng/µL. Microbial 16S libraries were prepared at the Plateforme Génome Transcriptome de Bordeaux, Bordeaux, France, by amplification and sequencing of the V3-V4 region of the 16S rRNA gene on an Illumina MiSeq machine, using a 2 × 250 base pair Illumina v2 kit. Quality filtering and processing were achieved with Cutadapt v4.0 and Python v3.9.9, USEARCH v11.0.667, and mothur v1.48.0 to identify 97% of the operational taxonomic units [44]. Abundance tables were imported and analyzed in R v4.1. The alpha and beta diversities were estimated using vegan 2.6-2 and compared using the Kruskal–Wallis and MANOVA tests, respectively. The indicator taxa were selected using the KW test and multiple testing correction with false discovery rate (FDR) correction. For the indicator taxa, an additional linear model was tested to determine the type of observed effect. Correlations between the relative abundance of the taxa and arbitrary values were tested using the Pearson correlation coefficient. Arbitrary units were allocated to the experimental groups, and six models were tested: (1) Totum-070 induces a return to normal that goes beyond the ND value (ND = 25, HFD = 75; HFD-T070 5% = 10); (2) Totum-070 induces a return to normal (ND = 25, HFD = 75; HFD-T070 5% = 25); (3) Totum-070 induces a partial return to normal (ND = 25, HFD = 75; HFD-T070 5% = 50); (4) Totum-070 does not rescue the changes induced by the HFD (ND = 25, HFD = 75; HFD-T070 5% = 75); (5) Totum-070 accentuates the HFD effect (ND = 25, HFD = 75; HFD-T070 5% = 100); and (6) Totum-070, but not the HFD, induces a change (ND = 100, HFD = 100; HFD-T070 5% = 10). The indicator taxa were identified by selecting taxa significantly associated with the supplementation group in the Kruskal–Wallis test, and for which at least one of the linear models was significant. If multiple linear models were significant, the model with the highest effect size was reported in Tables S2 and S3. Correlations between the indicator taxa and cholesterol levels were assessed using the Pearson correlation with FDR correction for multiple testing.

### 2.18. Statistical Analysis

Prism V.9.5.1 (GraphPad Software) was used for the statistic tests and figure drawing. Specifically, the Shapiro–Wilk normality test was used to determine whether the data followed a Gaussian distribution. If the data were not normally distributed, the Kruskal–Wallis nonparametric test was used, followed by the Dunn test for post hoc comparison. When the normal distribution was assumed, the data were compared with a one-way or two-way ANOVA and Tukey’s test for multiple comparisons. For measurements repeated over time, differences among the groups and time points were evaluated using a repeated-measure two-way ANOVA, followed by the Tukey’s post hoc test for multiple comparisons. If missing data did not allow using the repeated-measures 2-way ANOVA, a mixed-effects analysis was used. The values are presented as the mean ± SEM, unless specified otherwise. Differences were considered significant at *p* < 0.05.

## 3. Results

### 3.1. Supplementation with Totum-070 Reduces Hyperlipidemia and Improves Liver Steatosis and Inflammation in Hamsters without an Effect on Body Weight

To demonstrate Totum-070’s efficacy in a diet-induced hyperlipidemia model, the body weight- and fat mass-matched Syrian hamsters were fed the ND, HFD, or HFD with Totum-070 mixed at different concentrations: 3.5% (HFD-T070 3.5%), 4.25% (HFD-T070 4.25%), and 5% (HFD-T070 5%). The total caloric intake was not different between the ND and HFD groups, which could be explained by the significantly reduced food intake by the HFD animals compared with the ND animals (Table 2). Similarly, the body weights at the study’s end were similar between the ND and HFD groups (with/without supplementation), although the fat mass tended to increase, and the lean mass tended to decrease in the HFD groups (with/without supplementation) compared with the ND group (Table 2). Food intake was comparable in the three supplemented groups and the HFD group, demonstrating that Totum-070 was well tolerated at the tested concentrations. 

**Figure 1:**
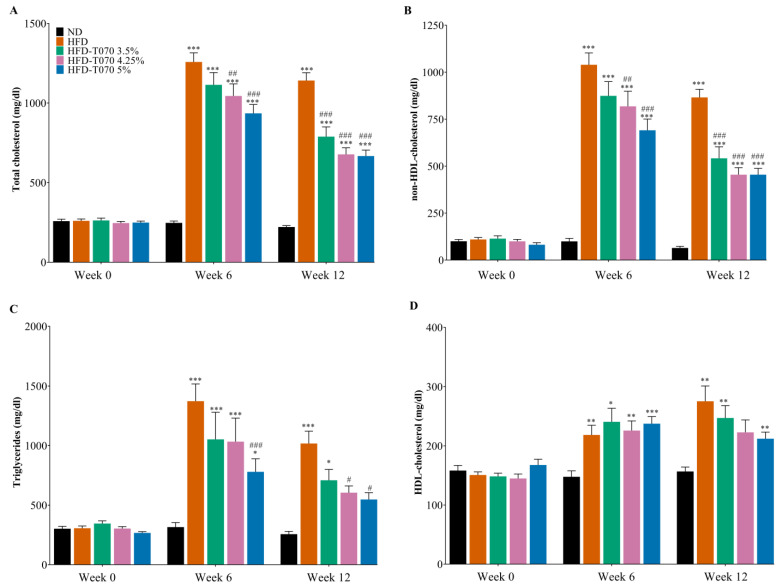
Supplementation with Totum-070 prevents hypercholesterolemia and hypertriglyceridemia in hamsters fed a high-fat diet. Male hamsters (*n* = 15/16 per group) were fed a normal diet (ND), high-fat diet (HFD), or HFD supplemented with Totum-070 at the following concentrations: 3.5% (HFD-T070 3.5%), 4.25% (HFD-T070 4,25%), and 5% (HFD-T070 5%). Blood samples were collected before the study’s start (week 0), during the study (week 6), and at the study’s end (week 12). (**A**) Concentration of total cholesterol; (**B**) non-HDL-cholesterol; (**C**) triglycerides; and (**D**) HDL-cholesterol in serum. * *p* < 0.05, ** *p* < 0.01, and *** *p* < 0.001 versus ND. # *p* < 0.05, ## *p* < 0.01, and ### *p* < 0.001 Totum-070 groups versus HFD. Data are the mean ± SEM.

Next, to determine Totum-070’s effect on the lipid profile, serum samples were collected before the study (week 0), during the study (week 6), and at the study’s end (week 12). As expected, dyslipidemia was observed in all the HFD groups, with a strong increase in the total cholesterol (Figure 1A), non-HDL-C (Figure 1B), and TG (Figure 1C), compared with the ND group at week 6 and week 12. However, their concentration was significantly lower in the three Totum-070 groups (in a dose-dependent manner) compared with the HFD group at week 6 and week 12. At week 12, the total cholesterol, non-HDL-C, and TG were reduced by 41% (*p* < 0.001), by 47% (*p* < 0.001), and by 46% (*p* < 0.05) in the HFD-T070 5% group compared with the HFD group. A slight increase in HDL-C was observed in the HFD groups compared with the ND group throughout the study; however, Totum-070 did not have any effect on the HDL-C level compared with the HFD group (Figure 1D). Altogether, these results demonstrate that Totum-070 has both hypocholesterolemic and hypotriglyceridemic properties.

**Figure 2:**
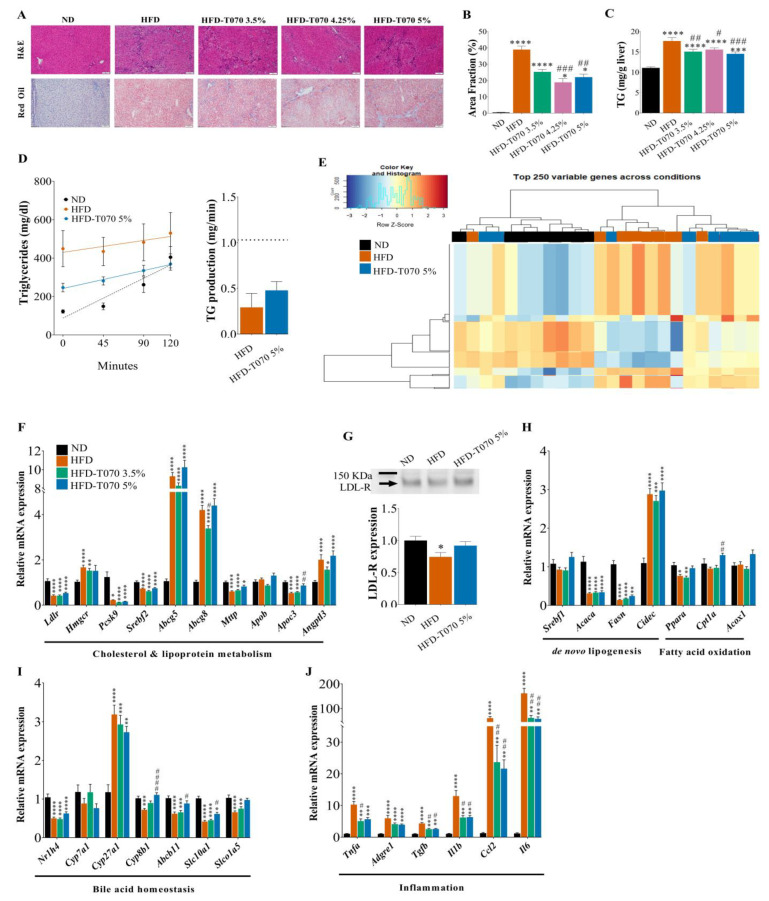
Effect of Totum-070 on liver lipid metabolism. (**A**) Paraffin-embedded sections of hamster livers stained with hematoxylin and eosin (H&E). Frozen sections of hamster livers stained with Oil Red O and counterstained with hematoxylin. Scale bars, 100 µm. (**B**) Quantification of Oil Red O in liver sections (at least three images per liver) shows reduction in neutral lipid accumulation in hamsters supplemented with Totum-070. (**C**) Triglyceride (TG) content in liver. (**D**) Changes in triglyceride concentration in serum samples collected at the indicated time points after injection of Poloxamer 407, and calculation of hepatic triglyceride (TG) production in HFD (*n* = 11) and HFD-T070 5% (*n* = 14) hamsters. An age-matched control group of hamsters (*n* = 6) was fed the ND. (**E**) Heatmap showing the top 250 genes differentially expressed (RNA-sequencing data) in hamster livers in the ND, HFD, and HFD-T070 5% groups (*n* = 8 per group) (**F**) Relative expression in liver of selected genes encoding proteins implicated in cholesterol and lipoprotein metabolism (*n* = 15/16 per group). (**G**) Immunoblot of LDL receptor expression in hamster liver samples from the indicated groups and quantification. (**H**) Relative expression in liver of selected genes encoding proteins implicated in lipid metabolism. (**I**) Relative expression in liver of selected genes encoding proteins implicated in bile acid metabolism. (**J**) Relative expression in liver of selected genes encoding proteins implicated in inflammation. * *p* < 0.05, ** *p* < 0.01, *** *p* < 0.001, and **** *p* < 0.00001 versus ND. # *p* < 0.05, ## *p* < 0.01, ### *p* < 0.001, and #### *p* < 0.00001 Totum-070 groups versus HFD. Data are the mean ± SEM.

Then, to assess Totum-070’s effect on liver lipid metabolism (Figure 2), liver tissue samples were stained with hematoxylin–eosin and Oil Red O (Figure 2A). After 12 weeks, the livers from the HFD groups displayed micro-vesicular steatosis of moderate to severe intensity and increased neutral lipid accumulation. These changes were reduced in the liver samples from the HFD-T070 3.5%, 4.25%, and 5% groups, as indicated by Oil Red O staining quantification (Figure 2B). Enzymatic assays in the liver extracts confirmed the decrease in hepatic TG content in the hamsters supplemented with Totum-070 compared with the HFD group (Figure 2C). An independent experiment was performed to assess the effect of Totum-070 on the hepatic TG output compared with HFD alone at week 12. Monitoring of the TG concentration in serum for 120 min following Poloxamer 407 injection, a nonionic detergent that inhibits lipoprotein lipase [34], showed that the liver TG secretion rate was similar in the HFD and HFD-T070 5% groups (Figure 2D).

To determine the liver metabolic adaption induced by Totum-070, the transcriptomes of livers from the ND, HFD, and HFD-T070 5% groups were analyzed by RNA sequencing (*n* = 8 per group). A data analysis revealed that 3434 genes were downregulated in the HFD group compared with the ND group and that 3567 genes were upregulated in the HFD group compared with the ND group. It should be noted that differentially expressed genes represented more than 50% of all the genes tested when comparing the two groups. In addition, 325 genes were downregulated and 186 genes were upregulated in the HFD-T070 5% group compared with the HFD group. The top 250 most variable genes in the three groups are shown in the heatmap in Figure 2E. This heatmap indicates that the ND and HFD groups were the most transcriptionally distinct, whereas the HFD-T070 5% group exhibited an intermediate profile. Among the top canonical pathways differentially regulated between the HFD and HFD-T070 5% groups (Table S1), the most important was the canonical metabolic pathway, and the implicated genes are listed in Table 3.

A detailed analysis of the expression of key regulators of hepatic lipid metabolism performed by RT-qPCR analysis (*n* = 15/16 per group) showed that the expression of *Ldlr* (encoding LDL receptor, a major actor of LDL removal from circulation) was drastically reduced in the livers from the HFD groups compared with the ND group, although a reduction was not observed in the HFD-T070 5% group (Figure 2F). This is consistent with the Western blot analysis (Figure 2G) showing that the LDL receptor was decreased by 26% in the HFD compared with the ND group (*p* < 0.05), and that its expression was nearly restored to normal level in the HFD-T070 5% group (*p* = 0.085 versus HFD). Other genes were also strongly affected by the HFD. Thus, *Abcg5* and *Abcg8* were upregulated in the HFD livers as expected because they are induced upon cholesterol accumulation in hepatocytes. Conversely, *Pcsk9* and *Srebf2* were downregulated in the livers from the HFD hamsters, in accordance with their repression upon the intracellular cholesterol level increase. However, no difference between the 3.5% and 5% Totum-070 groups and the HFD group was observed. *Fasn* and *Acaca* (genes implicated in de novo lipogenesis) were downregulated in the HFD group (Figure 2H), but this effect was slightly counteracted for *Fasn* in the 5% Totum-070 group (*p* = 0.11 compared to HFD). Similar results were obtained for *Mttp* and *Apoc3* (lipoprotein metabolism regulation), and for *Ppara*, *Cpt1a*, and *Acox1* (fatty acid oxidation) (Figure 2H). An analysis of the genes implicated in bile acid metabolism showed that the expression of *Cyp7a1*, which encodes the rate-limiting enzyme to convert cholesterol into bile acids in the liver, was similarly expressed in all the groups (Figure 2I). Conversely, secondary enzymes implicated in bile acid production pathways exhibited different expression patterns. Indeed, *Cyp27a1* was induced by the HFD, and Totum-070 did not have any effect (*p* > 0.99), while *Cyp8b1* was downregulated by the HFD, and its expression was restored by Totum-070 supplementation. Similarly, *Abcb11*, which encodes a bile acid transporter that promotes bile acid excretion into the bile duct, and *Slc10a1* and *Slco1a5*, transporters implicated in hepatocyte uptake of bile acids, were downregulated by the HFD, but their expression was restored by Totum-070 supplementation (Figure 2I). To further characterize Totum-070’s effects in the liver, the expression of inflammation markers (*Tnfα*, *Adgre1*, *Tgfβ*, *Il1β*, *Ccl2*, *Il6*) was investigated. Compared with the ND group, these markers were upregulated in the HFD group (Figure 2J), and supplementation with Totum-070 attenuated this effect. Collectively, these results indicate that Totum-070 tends to normalize the expression of liver genes altered by the HFD.

### 3.2. Totum-070 Modulates Intestinal Cholesterol Metabolism in Hamsters 

**Figure 3:**
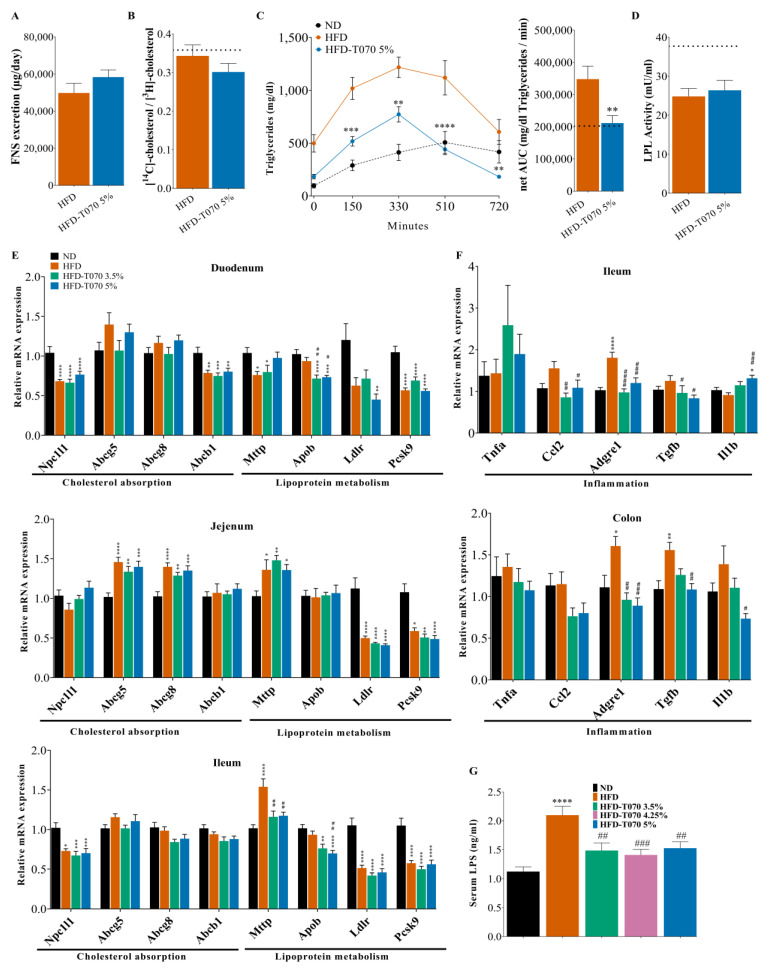
Effect of Totum-070 on intestinal lipid metabolism. (**A**) Excretion of fecal neutral sterols (FNS) quantified in fecal samples collected from day 75 to day 78. For each group (HFD and HFD-T070 5), pooled feces from 5 cages (*n* = 2/3 hamsters per cage) were used. (**B**) Results of the intestinal cholesterol absorption test performed after 12 weeks of supplementation in hamsters fed an HFD (*n* = 14) or HFD supplemented with T070 5% (*n* = 14). An age-matched control group of hamsters (*n* = 6) was fed the ND. (**C**) Changes in triglyceride concentration in serum after olive oil gavage, and calculation of the net area under the curve (AUC) in HFD (*n* = 13) and HFD-T070 5% (*n* = 13) hamsters after the oral fat tolerance test. An age-matched control group of hamsters (*n* = 6) was fed the ND. (**D**) Measurement of lipoprotein lipase (LPL) activity after heparin injection in HFD (*n* = 14) and HFD-T070 5% (*n* = 14) hamsters. An age-matched control group of hamsters (*n* = 6) was fed the ND. (**E**) Expression of genes encoding proteins implicated in lipid and lipoprotein metabolism in duodenum, jejunum and ileum. (**F**) Expression of genes encoding inflammation markers in ileum and colon. (**G**) Concentration of lipopolysaccharides (LPS) in the serum after 12 weeks. * *p* < 0.05, ** *p* < 0.01, *** *p* < 0.001, and **** *p* < 0.00001 versus ND. # *p* < 0.05, ## *p* < 0.01, ### *p* < 0.001, and #### *p* < 0.0001 (Totum-070 groups versus HFD). Data are the mean ± SEM.

To better understand Totum-070’s effect on cholesterol metabolism, neutral sterol excretion was quantified in fecal samples from the HFD and HFD-T070 5% hamsters collected from day 75 to day 78. The total fecal neutral sterol excretion was slightly higher (+17%; *p* = 0.22) in the HFD-T070 5% group than in the HFD group (Figure 3A), suggesting a decrease in intestinal cholesterol absorption during the study. To test this hypothesis, the intestinal cholesterol absorption was quantified in the HFD and HFD-T070 5% groups using a dual cholesterol isotope procedure [32,33]. A comparison of the [^14^C]/[^3^H] cholesterol ratio in the blood suggested a slight intestinal cholesterol absorption reduction (by 12%) in the HFD-T070 5% group (*p* = 0.25) (Figure 3B). The serum TG concentration was significantly higher in the HFD group than the HFD-T070 5% group at all time points (Figure 3C). For each group, the TG values were corrected to the baseline concentration (T0), and the net area under the curve (AUC) was calculated. The net AUC of serum TG was significantly reduced, by 39%, in the HFD-T070 5% group compared with the HFD group (*p* < 0.01). Conversely, the ex vivo post-heparin lipoprotein lipase activity assay results were not different between the HFD and HFD-T070 5% groups (Figure 3D), excluding any Totum-070 effect on TG hydrolysis in circulation. Altogether, these data strongly suggest that Totum-070’s TG-lowering action could be mediated through the inhibition of intestinal lipid absorption. 

Next, in order to thoroughly investigate Totum-070’s effects in the intestine, gene expression was analyzed in the different parts of the intestine. Totum-070 exposure for 12 weeks did not change the expression of the main players implicated in cholesterol absorption in the duodenum, jejunum, and ileum (Figure 3E). In the ileum, *Slc10a2*, encoding the ileal bile acid transporter protein (IBAT), was increased in the HFD group (*p* < 0.001) (Figure S1A), whereas its induction was slightly blunted in the HFD-T070 3.5% and HFD-T070 5% groups, although not significantly (*p* = 0.06 and *p* = 0.09 versus HFD, respectively). An analysis of the genes encoding various pro-inflammatory markers (*Ccl2*, *Adgre1*, *Tgfb*) in the ileum and colon indicated a reduction of inflammation in the HFD-T070 3.5% and HFD-T070 5% groups (Figure 3F). This observation was supported by the dosage of circulating LPS in the serum samples at the study’s end (Figure 3G). The serum LPS concentration was increased by 87.3% in the HFD (*p* < 0.0001) group compared with the ND group. Compared with the HFD group, the serum LPS concentration was significantly reduced by 29% (*p* < 0.01), 33% (*p* < 0.001), and 27% (*p* < 0.01) in the HFD-T070 3.5%, HFD-T070 4.25%, and HFD-T070 5% groups, respectively. 

### 3.3. Totum-070 Inhibits Cholesterol Uptake and Secretion in Enterocytes

**Figure 4:**
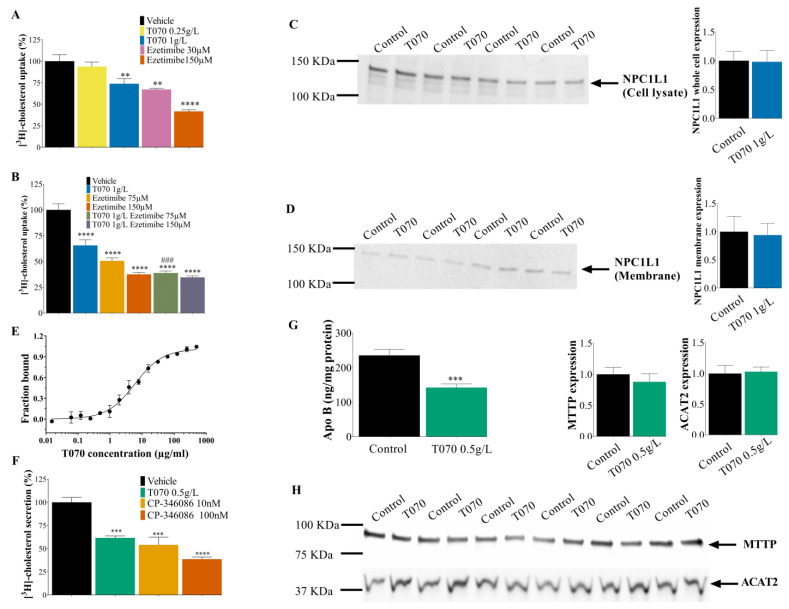
Totum-070 supplementation inhibits cholesterol uptake and secretion by differentiated human Caco-2 enterocytes. (**A**) Caco-2 cells differentiated on inserts were incubated (1 h) with [^3^H]-cholesterol-micelles at the apical pole with or without the presence of Totum-070 in the culture medium in the upper compartment. Ezetimibe was used as a positive control of [^3^H]-cholesterol uptake inhibition. Data are expressed as the percentage of control (vehicle). (**B**) Similar protocol as in (**A**) but combining Totum-070 and ezetimibe. The results suggest that Totum-070 may inhibit [^3^H]-cholesterol uptake in the same manner as ezetimibe. Data are expressed as the percentage of control (vehicle). (**C**,**D**) Immunoblot and quantification of the NPC1L1 transporter expression in whole-cell lysates (**C**) and membrane protein extracts (**D**) of Caco-2 cells. (**E**) Fraction of Totum-070 bound to NPC1L1-NTD, measured by MST, *n* = 2 for all points. Raw fluorescence (in counts) was normalized by expressing them as the fraction bound to the target. (**F**) Caco-2 cells differentiated on inserts were incubated with [^3^H]-cholesterol-micelles in the apical pole for 24 h before overnight incubation with or without the presence of Totum-070 in the culture medium in the upper compartment. CP-346086 was used as positive control of [^3^H]-cholesterol secretion inhibition. Data are expressed as the percentage of [^3^H]-cholesterol secretion in the basolateral medium in control cells (vehicle). (**G**) ApoB secretion in the basolateral medium from differentiated Caco-2 cells. (**H**) Immunoblot and quantification of MTTP and ACAT2 expression in differentiated Caco-2 cells. ** *p* < 0.01, *** *p* < 0.001, and **** *p* < 0.00001 versus vehicle. ### *p* < 0.001, Totum-070 1 g/L versus Totum-070 1 g/L + Ezetimibe 75 µM. Data are the mean ± SEM.

To determine Totum-070’s effect on cholesterol intestinal homeostasis, differentiated Caco-2 cells were incubated with [^3^H]-cholesterol with or without the presence of Totum-070 for 1 h, and then the [^3^H]-cholesterol uptake by the cells was measured. Ezetimibe was used as control because it blocks the NPC1L1, the cholesterol transporter protein. At 150 µM, ezetimibe reduced the [^3^H]-cholesterol uptake by 58% (*p* < 0.0001) compared with the vehicle (Figure 4A). This effect was not increased by using higher concentrations. Totum-070 significantly decreased the cholesterol uptake at 1 g/L (*p* < 0.01) (Figure 4A). As NPC1L1 inhibition might explain the reduction of [^3^H]-cholesterol uptake by Totum-070, the cells were incubated with [^3^H]-cholesterol, 150 µM ezetimibe, and/or 1 g/L Totum-070. On their own, Totum-070 (1 g/L) and ezetimibe (150 µM) decreased the cholesterol uptake by 34.5% (*p* < 0.001) and by 62.5%, respectively, but their combination did not further decrease the cholesterol uptake (Figure 4B). The combination of 75 µM ezetimibe, which reduced the cholesterol uptake by 49.4% on its own (*p* < 0.001), and 1 g/L Totum-070 led to a decrease in the cholesterol uptake by 61% compared with the control (*p* < 0.001) (Figure 4B). These findings suggest that NPC1L1 activity is required for Totum-070 inhibition of cholesterol uptake. A Western blot analysis of whole Caco-2 cell lysates (Figure 4C) and the membrane extracts (Figure 4D) of cultures treated for the cholesterol uptake analysis showed no effect of 1 g/L Totum-070 on NPC1L1 protein expression. The *N*-terminal domain of the NPC1L1 protein binds cholesterol and plays essential roles in cholesterol uptake [45]. Following the evidence that Totum-070 inhibited the NPC1L1 activity without changing its expression, we examined whether Totum-070 could interact with the *N*-terminus part of NPC1L1 through a direct binding event. Human recombinant NPC1L1-NTD was incubated with Totum-070 at concentrations ranging from 0.015 to 500 µg/mL, and the interactions were measured using microscale thermophoresis (MST). As detailed by Jerabek-Willemsen et al. [46], MST is a fluorescence-based technique to quantify an analysis of interactions between biomolecules, and its interest for characterizing the binding interaction between proteins and plant extracts has been validated previously [47]. Here, we demonstrate that NPC1L1-NTD interacted with Totum-070 with a dissociation constant (K_d_) of 5.934 µg/mL (Figure 4E). 

Re-packaging of intracellular esterified cholesterol and secretion into chylomicrons is another mechanism that influences intestinal cholesterol absorption. To assess Totum-070’s effect on cholesterol secretion, differentiated Caco-2 cells were incubated with [^3^H]-cholesterol for 24 h prior to overnight incubation with Totum-070, followed by [^3^H]-cholesterol quantification in the basolateral medium. Incubation with 0.5 g/L Totum-070 led to a reduction of 38.4% of cholesterol secretion compared with control (*p* < 0.001) (Figure 4F). Incubation with CP-346086, an inhibitor of MTTP enzymatic activity used as a positive control, decreased cholesterol secretion by 46% at 10 nM (*p* < 0.001) and by 61% at 100 nM (*p* < 0.0001) (Figure 4F). Consistent with the inhibition of cholesterol secretion, ApoB secretion in the basolateral medium was also reduced by 0.5 g/L Totum-070 (−39.5% compared with control, *p* < 0.001) (Figure 4G). Of note, a Western blot analysis of the Caco-2 cells after overnight incubation with 0.5 g/L Totum-070 showed no effect on the expression level of MTTP and ACAT2, two major enzymes controlling the secretion of esterified cholesterol (Figure 4H). Altogether, these results demonstrate that Totum-070 inhibits intestinal cholesterol absorption, including cholesterol uptake and chylomicron secretion.

### 3.4. Totum-070 Influences Gut Microbiota in Hamsters

**Figure 5:**
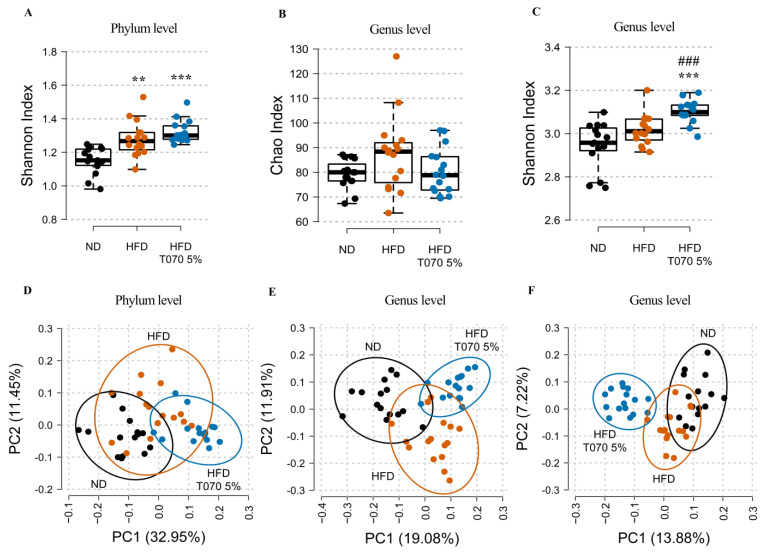
Effect of Totum-070 on cecal microbiota richness, diversity, and composition. (**A**) Shannon diversity index at the phylum level. (**B**) Rarefied Chao index (index of richness) at the genus level. (**C**) Shannon diversity index at the genus level. (**D**,**E**) Principal coordinate analyses based on the Bray–Curtis dissimilarity index at the phylum (**D**) and genus (**E**) levels, respectively, with 95% confidence ellipses. (**F**) Principal coordinate analysis based on the Jaccard dissimilarity index at the genus levels, with 95% confidence ellipses. Boxplots show the median, 25th, and 75th percentiles and adjacent values (*n* = 15/16 per group). ** *p* < 0.01 and *** *p* < 0.001 versus ND. ### *p* < 0.001 Totum-070 5% group versus HFD.

Whole 16S rRNA gene sequencing of the cecum content was used to assess the impact of the ND, HFD, and HFD supplemented with 5% Totum-070 on the intestinal microbiota composition at the study’s end. At the phylum level, evenness was significantly increased in the HFD (*p* < 0.01) and HFD-T070 5% (*p* < 0.001) groups compared with the ND group (Figure 5A), without any difference between HFD and HFD-T0710 5%. At the genus level, species richness was similar in the three groups (Figure 5B). Conversely, evenness was increased in the HFD-T070 5% group compared with both the HFD (*p* < 0.001) and ND groups (*p* < 0.001) (Figure 5C). A principal coordinate analysis of the relative abundance of intestinal microbial communities at the phylum level using the Bray–Curtis dissimilarity index followed by PERMANOVA showed that the microbial composition differed significantly among the three groups (Bray–Curtis, adonis: *p* < 0.0001 for all the groups, R_NDvsHFD_ = 0.24, R_NDvsHFD-T0705%_ = 0.60, R_HFDvsHFD-T0705%_ = 0.31) (Figure 5D). The same analysis at the genus level confirmed that the HFD strongly affected the microbial composition in the cecum compared with the ND (Bray–Curtis, adonis: *p* < 0.0001, R_NDvsHFD_ = 0.26 (Figure 5E) and Jaccard, adonis: *p* < 0.0001, R_NDvsHFD_ = 0.12 (Figure 5F)). The microbial composition in the HFD-T070 5% group differed from that of the ND group (Bray–Curtis, adonis: *p* < 0.0001 R_NDvsHFD-T0705%_ = 0.36 (Figure 5E) and Jaccard, adonis: *p* < 0.0001, R_NDvsHFD-T0705%_ = 0.25 (Figure 5F)), as well as the HFD group (Bray–Curtis, adonis: *p* < 0.0001, R_HFDvsHFD-T0705%_ = 0.21 (Figure 5E) and Jaccard, adonis: *p* < 0.0001, R_HFDvsHFD-T0705%_ = 0.17 (Figure 5F)). This highlighted a stronger impact of the HFD with Totum-070 supplementation than HFD alone at the genus level. 

Given the strong effect on the alpha and beta diversity measures in the HFD-T070 5% group, and the visible differences in the average composition at the phylum and genus levels (Tables S2 and S3, respectively), an indicator species analysis was performed to identify the taxa specifically affected by the diet. At the phylum level, HFD significantly decreased the abundance of Firmicutes (−15%, *p* < 0.01) and Actinobacteria (−57%, *p* < 0.01) compared with ND (Table S2) and increased the abundance of Campylobacterota, Deferribacteres, and Proteobacteria (+211%, *p* < 0.05; +123%, *p* < 0.05; and +44%, *p* < 0.01 vs. ND, respectively). The Firmicutes/Bacteroidetes ratio tended to decrease in the HFD group (−15%; not significant) compared with the ND group (Table S2). Supplementation with 5% Totum-070 accentuated the reduction in Firmicutes abundance compared with the ND group (−34%, *p* < 0.0001) and the HFD group (−23%, *p* < 0.05). Conversely, the abundance of Bacteroidetes was higher in the HFD-T070 5% group than the HFD group (+23%, *p* < 0.001). Therefore, the Firmicutes/Bacteroidetes ratio further decreased in the HFD-T070 5% group compared with the ND (−47%, *p* < 0.001) and HFD (−37%, *p* < 0.001) groups (Table S2). At the genus level (Table S3), 39 genera were significantly different between at least two groups. An analysis of the correlation of the abundance of these genera with the total cholesterol level in each animal at the study’s end showed that 14 of the genera influenced by Totum-070 were correlated with the total cholesterol level (Table S3). Specifically, the abundance of uncl. *Muribaculaceae*, *Duncaniella*, and uncl. *Desulfovibrionaceae* was inversely correlated with the cholesterol level. These genera were decreased in the HFD group compared with the ND group (−37%, *p* < 0.01; −37%, ns and −32%, ns, respectively). Supplementation with Totum-070 significantly increased their abundance compared with the HFD group (+52%, *p* < 0.01; +45%, *p* < 0.05; and +54%, *p* < 0.05, respectively) and restored their abundance to the level observed in the ND group. Among the genera the abundance of which was positively correlated with the cholesterol level, *Lactococcus* and *Flintibacter* were higher in the HFD group than the ND group (+120%, ns and +59%, *p* < 0.05, respectively). In the HFD-T070 5% group, their abundance was reduced compared with HFD (−55%, *p* < 0.05 and −29%, *p* < 0.05) to levels comparable to those in the ND group. Altogether, HFD-T070 5% strongly affected the gut microbial composition and influenced the abundance of several cholesterol-related genera in correlation with its hypocholesterolemic action.

## 4. Discussion

This study demonstrated the efficacity of Totum-070, a polyphenol-rich mixture, to reduce total cholesterol, non-HDL cholesterol, and triglyceride levels in a hamster model of diet-induced dyslipidemia and identified in vivo and in vitro pathways through which Totum-070 prevents hyperlipidemia. This dose-dependent cholesterol reduction was observed from week 6 of supplementation until the study’s end (week 12). Importantly, most of the total cholesterol lost was non-HDL-C (Figure 1D), a particularly interesting result, given the link between elevated plasma non-HDL-C and ASCVD [48,49]. The hypocholesterolemic effect of Totum-070 is independent of the modulation of caloric intake and body weight (Table 2). Besides its beneficial effect on serum cholesterol and triglycerides, supplementation with Totum-070 reduced triglyceride accumulation in liver (Figure 2C). Signs of hepatic steatosis were clearly identified in the HFD group but were reduced in the groups receiving HFD with Totum-070. Moreover, Totum-070 had a systemic anti-inflammatory effect by preventing elevation of serum LPS (Figure 3G) and counteracting the induction of pro-inflammatory genes in the liver (Figure 2J) and intestine (Figure 3F). These results strengthened Totum-070’s role in the prevention of cardiometabolic disorders commonly associated with hyperlipidemia [50,51]. 

Totum-070 combines five plant extracts selected to act on different organs involved in the regulation of cholesterol homeostasis. The effect on dyslipidemia of several of these plant extracts and compounds present in Totum-070 has already been studied in animal models, such as the hypolipidemic effect of artichoke leaf extracts in rats fed an HFD [14,52,53], the beneficial action of olive leaf extracts in HFD-mice [54] and rats [18,55], and hyperlipemia reduction by Goji berries [21,24] and black pepper extracts [25] in rats and mice. The choice of animal model in the present work is also fundamental. Indeed, Syrian hamsters better mimic cholesterol metabolism and the enzymatic pathways involved in human-like lipoprotein disorders, compared with other rodent models that are naturally resistant to diet-induced hypercholesterolemia. Their receptor-mediated uptake of LDL-C, cholesteryl ester transfer protein activity, and hepatic ApoB-100 and intestinal ApoB-48 secretion, comparable to those described in humans [56,57], allow for studying the effect of a food supplement on HFD-induced dyslipidemia. Plasma cholesterol levels depend on several parameters, including endogenous cholesterol synthesis, cholesterol removal from circulation, dietary cholesterol absorption, and cholesterol reabsorption from bile. The intestine and liver play central roles in cholesterol homeostasis and, consequently, in hypercholesterolemia and cardiovascular risk. In this study, an HFD’s effects on lipid and lipoprotein metabolism in the liver and intestine were investigated to understand Totum-070’s mechanisms of action. A gene expression analysis of the main regulators for cholesterol (Figure 2F) and fatty acid (Figure 2H) metabolism in the liver showed no to scarce effects of Totum-070 compared to HFD-fed hamsters. An investigation of bile acid homeostasis depicted a normalization of the transcripts altered upon HFD with the 5% Totum-070 supplementation. LDL-C is removed from plasma predominantly by an LDL receptor, a transmembrane protein expressed at the plasma membrane of liver cells and which is responsible for LDL particle internalization via receptor-mediated endocytosis [58]. Therefore, regulation of the liver LDL receptor is a key mechanism by which therapeutic agents can decrease cholesterol levels. Previous reports documented LDL receptor upregulation in cultured hepatocytes when cells were incubated with piperine [59] or hydroxytyrosol [60], two biomolecules present in Totum-070 (Table 1). In our study, liver LDL receptor expression was reduced by 26% in the HFD group, but in the HFD-T070 5% group, its expression was restored to the level observed in the ND group (Figure 2G). This observation may partly explain the reduction of non-HDL-C in HFD-fed hamsters supplemented with Totum-070. In addition, LDL receptor implication in the production of newly synthetized triglyceride-rich particles in the liver is a mechanism that regulates the hepatic triglyceride output [61,62]. Modulation of the liver’s secretion of triglyceride-rich particles might partly explain Totum-070’s hypotriglyceridemic effect. However, the livers’ triglyceride production was similar in the Totum-070-supplemented groups and in the HFD group (Figure 2D). The activity of lipoprotein lipase, which mediates the hydrolysis of circulating triglycerides, was also comparable between the groups (Figure 3D). Conversely, the oral fat tolerance test showed that the amplitude and duration of postprandial hypertriglyceridemia were significantly reduced in the Totum-070 group compared with the HFD group (Figure 3C). This is an important result because postprandial hyperlipidemia is a cardiovascular risk factor, especially in patients with metabolic syndrome [63]. As no difference was observed in the lipoprotein lipase activity, Totum-070 might lower postprandial hypertriglyceridemia by decreasing the release of intestinal triglyceride-rich lipoproteins. Several biomolecules present in Totum-070 (i.e., piperine [64] and luteolin [65,66]) have been previously reported to modulate intestinal lipid metabolism, notably through the inhibition of NPC1L1 expression and trafficking to the cellular membrane. This transmembrane protein localized at the apical membrane of enterocytes is involved in cholesterol uptake by enterocytes. As suggested by the effect of ezetimibe, an NPC1L1 inhibitor on postprandial hypertriglyceridemia [67], NPC1L1 inhibition may also lead to the reduction in intestinal triglyceride production [67] Totum-070 reduced cholesterol uptake in vitro, and this effect was blunted by co-incubation with ezetimibe (Figure 4B). This suggests that NPC1L1 activity is required for a Totum-070-mediated reduction of cholesterol uptake. Totum-070’s effect was not due to the regulation of NPC1L1 expression or location at the cell membrane (Figure 4C,D), but possibly through the inhibition of NPC1L1 activity. The structure of NPC1L1 or the uptake route may have been altered because cholesterol uptake by Caco-2 cells was inhibited by the Totum-070 bioactive molecules within 1 h of incubation. For example, ezetimibe binding to the middle extracellular domain of NPC1L1 causes conformational changes in the NPC1L1 protein to disturb NPC1L1 and cholesterol interactions [68]. The *N*-terminus part of NPC1L1 contains the sterol-binding domain that is essential for extracellular cholesterol binding to NPC1L1 and uptake into the cell [45]. Using MST, we demonstrated direct binding of Totum-070 to the *N*-terminal domain of the NPC1L1 protein (Figure 4E). Thus, we could speculate that Totum-070’s interaction with NPC1L1-NTD would suppress cholesterol binding to NPC1L1, resulting in the inhibition of NPC1L1 transport. Totum-070 also interfered with cholesterol secretion into newly synthetized particles in vitro (Figure 4F). The exact molecular mechanisms need to be elucidated, because Totum-070 did not have any effect on MTTP or ACAT2 expression in the Caco-2 cells (Figure 4H). This reduction in cholesterol uptake in the intestine was confirmed in vivo, as fecal neutral sterols were increased by 17% in hamsters supplemented with Totum-070 compared with the HFD group (Figure 3A). In line with the increased cholesterol excretion in the feces, dietary cholesterol absorption in the intestine was decreased in the Totum-070-supplemented groups (Figure 3B). Altogether, these results demonstrate that intestinal cholesterol absorption was reduced by Totum-070 supplementation, suggesting that chronic supplementation with Totum-070 (12 weeks) might contribute to decrease cholesterol in this model. At the end, our data show that Totum-070 supplementation is an effective strategy to reduce intestinal cholesterol absorption.

The gut microbiota has emerged as a crucial factor that influences cholesterol metabolism [69,70], and many studies have already linked the imbalance or maladaptation of the microbial community, defined as “dysbiosis”, to host lipid metabolism and metabolic disorders [71,72]. A meta-analysis found that the Firmicutes to Bacteroidetes ratio is increased in HFD-fed mice compared with ND-fed mice [73]. Importantly, an increase in the Firmicutes to Bacteroidetes ratio has been associated with cardiovascular risk factors, such as obesity [71] and hypertension [72]. Moreover, this ratio has been significantly correlated with the circulating total cholesterol concentration in mice [74] and hamsters [75]. In our study, the Firmicutes to Bacteroidetes ratio did not change significantly between the ND group and HFD group but decreased significantly in the Totum-070-supplemented groups compared with the HFD group (Table S2). Moreover, uncl. *Muribaculaceae*, *Duncaniella*, and uncl. *Desulfovibrionaceae*, which were negatively correlated with the cholesterol level, decreased in the HFD group and were restored to normal levels upon Totum-070 supplementation (Table S3). The abundance of *Duncaniella,* which has anti-inflammatory properties [76], reduced in western diet-fed mice [74], and is negatively correlated with LDL-cholesterol [77], as observed in the present study. Similarly, the *Muribaculaceae* community was significantly reduced in the intestinal flora of HFD mice [78], whereas its increase might contribute to the serum cholesterol-lowering effect of plant proteins in dyslipidemic hamsters [75]. *Muribaculaceae* may also be a potential biomarker of cholesterol reduction [79]. Moreover, our study shows that the abundance of *Lactococcus* and *Flintibacter* positively correlated with total cholesterol level (Table S3). *Lactococcus* and *Flintibacter* were more abundant in the HFD group compared with the ND group, but supplementation with Totum-070 reduced their abundances to the level observed in the ND group. Consistent with our observation, previous reports described increased *Lactococcus* [80] and *Flintibacter* [81] in animal models fed an HFD. Altogether, the analysis of the cecal microbiota showed alterations of the microbial balance in the HFD group compared with the ND group. Our results suggest that the Totum-070-supplemented group displayed an alternative profile compared with the HFD and ND groups. Some specific taxa were influenced by Totum-070 in correlation with its beneficial effect on cholesterol lowering. However, it remains to be clarified whether these changes are a cause or a consequence of the hypocholesterolemic effect.

This study has potential limitations. It has been shown that the lipid profile differs by sex, with estrogen providing protection in non-menopausal women [82,83]. In this study, we exclusively used male hamsters; therefore, future studies could explore the effect of Totum-070 on females. Regarding NPC1L1, this work explored its role in Totum-070’s intestinal effects. However, new studies in genetically modified mice lacking NPC1L1 [84,85] would complement the understanding of Totum-070’s cholesterol-lowering mechanism. Additionally, the actions of Totum-070 were limited to lipid homeostasis, and the potential effects of Totum-070 in preventing or delaying the atherosclerotic process were not covered in this study. Although hamsters exhibit metabolic similarities more comparable to humans than other rodents, they do not develop aortic atherosclerotic lesions [86]. Therefore, the use of a suitable preclinical model prone to atherosclerosis development, such as LDL receptor- or ApoE-deficient mice [86], will be required to evaluate the beneficial effects of Totum-070 on atherosclerosis. Finally, no adverse effects from Totum-070 supplementation were observed in our animal studies. To our knowledge, only minor to no gastrointestinal effects have been reported in trials using plant extracts comprising Totum-070, such as olive leaf extracts [87] or artichoke leaf extracts [88]. Future high-quality clinical studies will need to confirm the hypocholesterolemic potential of this formulation in humans and document its safety and tolerance.

## 5. Conclusions

In conclusion, we demonstrated that Totum-070 can reduce hypercholesterolemia in a hamster model of diet-induced dyslipidemia. Furthermore, the HFD-induced hypertriglyceridemia and hepatic triglyceride accumulation were reduced by Totum-070 supplementation. In vivo and in vitro analyses in hamsters and human enterocytes, respectively, demonstrated that Totum-070 directly inhibits intestinal cholesterol absorption potentially through its interaction with the *N*-terminal domain of NPC1L1. The additional beneficial effects observed on the gut microbiota composition also might contribute to Totum-070’s mode of action, making Totum-070 an innovative natural ingredient to prevent blood cholesterol elevation, a major risk factor for ASCVD.

## Figures and Tables

**Table 1:** Chemical characterization of Totum-070.

Compound Type (Sorted by Families)	Totum-070 Content (*w*/*w*)
Total sugars	41.35
Total lipids	7.30
Total proteins	2.05
Betaine	0.23
Total phenolic compounds	15.42
Mono-caffeoylquinic acids	
Chlorogenic acid	0.81
Cryptochlorogenic acid	0.01
Other mono-caffeoylquinic acids	0.25
Dicaffeoylquinic acids	
Cynarin	0.16
3-5 dicaffeoylquinic acid	0.18
4-5 dicaffeoylquinic acid	0.14
Other dicaffeoylquinic acids	0.11
Caffeic acid	0.01
Oleuropein	4.6
Oleuropein isomers	0.47
Hydroxytyrosol	0.08
Luteolin	0.01
Luteolin-7-O-glucoside	0.27
Luteolin-7-O-glucoside isomer	0.02
Luteolin-4-O-glucoside	0.06
Luteolin-7-O-glucuronide	0.29
Apigenin-7-O-glucoside	0.03
Apigenin-7-O-glucuronide	0.17
Apigenin-6-*C*-glucoside-8-*C*-arabinoside (Shaftoside)	0.01
Apigenin-6,8-*C*-diglucoside (Vicenin 2)	0.02
Apigenin-7-O-rutinoside	0.01
Eriodictyol-7-O-glucoside	0.04
Flavanomarein	0.14
Marein	0.05
Maritimein	0.05
Rutin	0.02
Verbascoside	0.08
Terpenes and terpenoids	
Oleanolic acid	1.64
Saponins	
Chrysanthellin A	0.28
Chrysanthellin B	0.27
Iridoids	
Oleoside	0.01
Cynaropicrin	0.07
Alkaloids	
Piperine	0.06

**Table 2:** Effect of the different diets on body weight, body composition, food intake, and caloric intake.

Diet Supplementation	ND -	HFD -	HFD T070 3.5%	HFD T070 4.25%	HFD T070 5%
Final body weight (g)	129.2 ± 2.6	124.4 ± 2.7	124.8 ± 2.6	126.5 ± 3.3	123.4 ± 1.2
Final fat mass (g)	18.12 ± 1.3	20.14 ± 0.6	22.79 ± 1	23.36 ± 1 *	22.26 ± 1
Final lean mass (g)	106.3 ± 2.2	98.61 ± 2.3	97.17 ± 1.9 *	98.74 ± 2.7	96.78 ± 1.8 *
Cumulative food intake (g)	436 ± 7.1	375 ± 11.5 **	386 ± 10.5	401.5 ± 8	397 ± 15.4
Cumulative caloric intake (Kcal)	1680 ± 27.3	1688 ± 52	1700 ± 46.3	1766 ± 35.2	1748 ± 68

Hamsters were fed a normal diet (ND, *n* = 15), a high-fat diet (*n* = 16), or a high-fat diet supplemented with Totum-070 at the indicated concentration (T070 3.5%, T070 4.25% and T070 5%, *n* = 16 per group) for 12 weeks. * *p* < 0.05 and ** *p* < 0.01 versus ND. Data are the mean ± SEM.

**Table 3:** Genes that belong to the canonical metabolic pathway and that are differentially expressed in HFD hamster livers compared with HFD-T070 5% hamster livers (*n* = 8 per group).

Log Fold-Change	*p*-Values HFD	Golden Hamster Gene ID	Gene Name	Protein
2.43	0.001509	ENSMAUG00000000351	*Lipg*	lipase G, endothelial type
2.39	0.017545	ENSMAUG00000000465	*Acly*	ATP-citrate synthase
2.24	0.000089	ENSMAUG00000001919	*Atp6v0d2*	V-type proton ATPase subunit d 2
2.08	0.000001	ENSMAUG00000008803	*Alpl*	alkaline phosphatase
2.06	0.000125	ENSMAUG00000004155	*Asns*	asparagine synthetase
1.51	0.000422	ENSMAUG00000009435	*Bst1*	ADP-ribosyl cyclase 2
1.47	0.001238	ENSMAUG00000005505	*Mboat2*	lysophospholipid acyltransferase 2
1.35	0.002228	ENSMAUG00000018983	*Alox5*	polyunsaturated fatty acid 5-lipoxygenase
1.21	0.000716	ENSMAUG00000001135	*B3galnt1*	UDP-GalNAc:beta-1,3-*N*-acetylgalactosaminyltransferase 1
1.18	0.000011	ENSMAUG00000000598	N/A	N/A
1.14	0.000212	ENSMAUG00000009261	*Mthfd2*	NAD-dependent methylene tetrahydrofolate dehydrogenase cyclohydrolase
1.14	0.000757	ENSMAUG00000017913	*Mboat1*	lysophospholipid acyltransferase 1
1.07	0.003228	ENSMAUG00000020920	*Hk2*	hexokinase-2
1.07	0.001393	ENSMAUG00000007951	*Elovl7*	elongation of very long chain fatty acids protein 7
1.02	0.000411	ENSMAUG00000016079	*Acss1*	acetyl-coenzyme A synthetase 2-like, mitochondrial
1.01	0.002529	ENSMAUG00000004073	*Sqle*	squalene monooxygenase
1.00	0.001829	ENSMAUG00000019624	*Pla2g7*	platelet-activating factor acetylhydrolase
−1.02	0.005813	ENSMAUG00000003438	*Gck*	hexokinase-4

## Data Availability

The raw RNA sequencing data from the hamster livers has been deposited in GEO under BioProject PRJNA945002 with the accession number GSE227411 (BioSamples: GSM7100119 to GSM7100142). The raw 16S rRNA gene sequencing data from hamster cecal microbiota has been deposited in the Sequence Read Archive under BioProject PRJNA549583 with the following accession numbers: SRR22824159 to SRR22824205 (BioSamples: SAMN32320356 to SAMN32320402). All the remaining data described in the manuscript are shown in the manuscript and Supplementary Data.

## Supplementary Materials

The following supporting information can be downloaded at: https://www.mdpi.com/article/10.3390/nu15245056/s1, Figure S1: expression of genes encoding proteins implicated in bile acid homeostasis in ileum; Table S1: list annotation of differentially expressed hepatic genes within KEGG pathways between HFD and HFD T070 5% groups using the *Cricetulus griseus*’ ortholog gene identifiers; Table S2: cecal microbiota: indicator phyla. Indicator taxa were identified as those the abundance of which differed significantly in ≥2 groups; Table S3: cecal microbiota: indicator genera. Indicator taxa were identified as those whose abundance differ significantly between two groups or more.; Table S4: List of hamster primers used for qPCR.

## Abbreviations

ACAT2	acetyl-CoA acetyltransferase 2
ASCVD	Atherosclerotic cardiovascular disease
AUC	Area under the curve
FNS	Fecal neutral sterols
HDL-C	High-density lipoprotein cholesterol
HFD	High-fat diet
HTPT	Hepatic triglycerides production test
IBAT	Ileal bile acid transporter
LDL-C	Low-density lipoprotein cholesterol
LPL	Lipoprotein lipase
LPS	Lipopolysaccharides
MTTP	Microsomal triglyceride transfer protein
MST	Microscale thermophoresis
ND	Normal diet
NTD	N-terminal domain
NPC1L1	Niemann-pick C1-like 1
OFTT	Oral fat tolerance test
TG	Triglycerides
VLDL	Very low-density lipoprotein
