# Supplementary material for: Totum-070, a Polyphenol-Rich Plant Extract, Prevents Hypercholesterolemia in High-Fat Diet-Fed Hamsters by Inhibiting Intestinal Cholesterol Absorption
Source: Nutrients. 2023 Dec 9;15(24):5056. doi: 10.3390/nu15245056 (PMC10746001; doi:10.3390/nu15245056)
Supplement: Supplementary file 1 [file nutrients-15-05056-s001.zip › Figure S1.pdf]

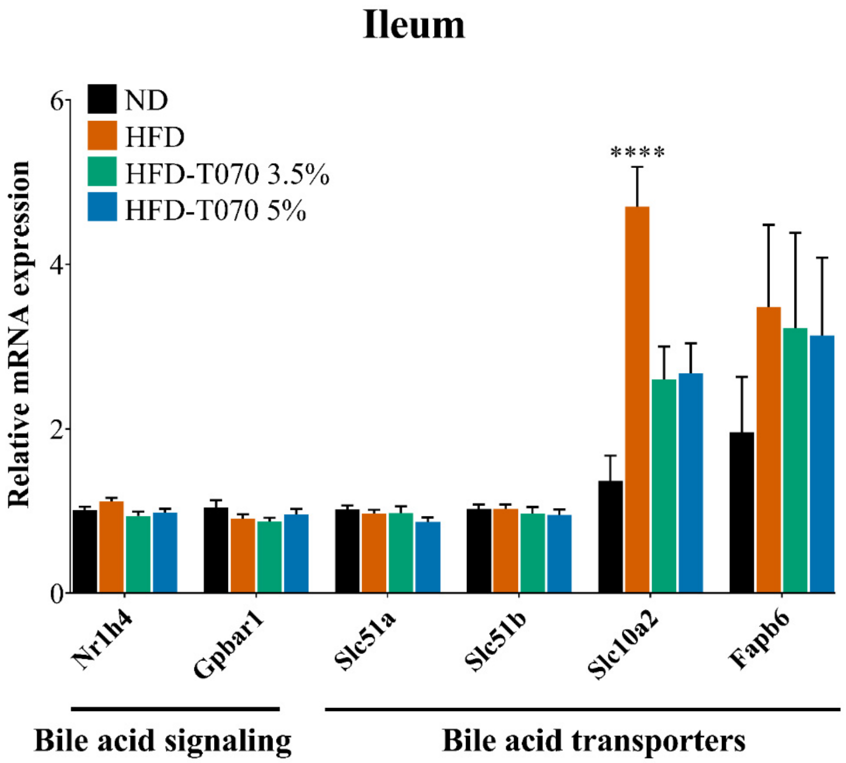

Figure S1. Expression of genes encoding proteins implicated in bile acid homeostasis in ileum. **** p <0.0001 *versus* ND. Data are the mean ± SEM
