# Supplementary material for: Totum-070, a Polyphenol-Rich Plant Extract, Prevents Hypercholesterolemia in High-Fat Diet-Fed Hamsters by Inhibiting Intestinal Cholesterol Absorption
Source: Nutrients. 2023 Dec 9;15(24):5056. doi: 10.3390/nu15245056 (PMC10746001; doi:10.3390/nu15245056)
Supplement: Supplementary file 1 [file nutrients-15-05056-s001.zip › Table S1.pdf]

Table S1. List annotation of differentially expressed hepatic genes within KEGG pathways between HFD and HFD T070 5% groups using the *Cricetulus griseus'* ortholog gene identifiers.

Pathway	Gene count	Fold enrichment	p values
cge01100:Metabolic pathways	19	1.47	0.0744
cge05010:Alzheimer disease	11	3.10	0.0024
cge05022:Pathways of neurodegeneration	11	2.60	0.0081
cge05012:Parkinson disease	9	3.94	0.0017
cge05020:Prion disease	8	3.49	0.0071
cge05014:Amyotrophic lateral sclerosis	8	2.63	0.0299
cge05152:Tuberculosis	7	4.62	0.0037
cge05208:Chemical carcinogenesis – reactive oxygen species	7	3.11	0.0232
cge05016:Huntington disease	7	2.74	0.0397
cge04932:Non-alcoholic fatty liver disease	6	4.53	0.0097
cge04140:Autophagy - animal	6	4.19	0.0133
cge04621:NOD-like receptor signaling pathway	6	4.15	0.0139
cge05415:Diabetic cardiomyopathy	6	3.36	0.0310
cge04714:Thermogenesis	6	2.66	0.0715
cge05163:Human cytomegalovirus infection	6	2.57	0.0799
cge04625:C-type lectin receptor signaling pathway	5	4.38	0.0259
cge05418:Fluid shear stress and atherosclerosis	5	4.24	0.0288
cge04723:Retrograde endocannabinoid signaling	5	4.16	0.0306
cge04722:Neurotrophin signaling pathway	5	4.01	0.0344
cge04371:Apelin signaling pathway	5	3.46	0.0542
cge04024:cAMP signaling pathway	5	2.93	0.0876
cge03050:Proteasome	4	11.36	0.0050
cge04924:Renin secretion	4	7.26	0.0169
cge05133:Pertussis	4	7.06	0.0182
cge04925:Aldosterone synthesis and secretion	4	5.28	0.0386
cge04728:Dopaminergic synapse	4	3.71	0.0904
cge04936:Alcoholic liver disease	4	3.60	0.0963
