# Supplementary material for: Totum-070, a Polyphenol-Rich Plant Extract, Prevents Hypercholesterolemia in High-Fat Diet-Fed Hamsters by Inhibiting Intestinal Cholesterol Absorption
Source: Nutrients. 2023 Dec 9;15(24):5056. doi: 10.3390/nu15245056 (PMC10746001; doi:10.3390/nu15245056)
Supplement: Supplementary file 1 [file nutrients-15-05056-s001.zip › Table S2.pdf]

Table S2. Cecal microbiota: indicator phyla. Indicator taxa were identified as those the abundance of which differed significantly in ≥2 groups. Relative abundances are presented as the mean ± SD. and p values were adjusted for multiple testing. n=15/16 per group. F/B: Firmicutes/Bacteroidetes.

	% of relative abundance			Adjusted p values		
**Phylum**	**ND**	**HFD**	**HFD-T070 5%**	**ND vs HFD**	**ND vs HFD-T070 5%**	**HFD vs HFD T070 5%**
Actinobacteria	0.49±0.21	0.21±0.07	0.22±0.09	p=0.0018	p =0.0019	p =0.5651
Elusimicrobia	0.06±0.06	0.17±0.17	0.19±0.17	p=0.0591	p =0.0129	p =0.5970
Firmicutes	49.46±5.15	42.14±5.56	32.55±4.33	p =0.0018	p =0.0001	p =0.0194
Campilobacterota	0.17±0.15	0.53±0.45	0.85±0.76	p =0.0455	p =0.0001	p =0.3344
Deferribacteres	0.26±0.15	0.58±0.33	0.90±0.53	p =0.0107	p =0.0001	p =0.1000
Proteobacteria	10.23±2.24	14.74±2.70	14.54±1.52	p =0.0018	p =0.0001	p =0.5651
Bacteroidetes	34.63±3.75	34.94±5.15	42.90±4.76	p =0.6353	p =0.0002	p =0.0004
uncl. Bacteria	3.99±1.11	4.55±1.20	6.83±1.99	p =0.4956	p =0.0001	p =0.0023
F/B ratio	1.46±0.32	1.24±0.29	0.78±0.19	p =0.0753	p =0.0000	p =0.0001
