# Supplementary material for: Totum-070, a Polyphenol-Rich Plant Extract, Prevents Hypercholesterolemia in High-Fat Diet-Fed Hamsters by Inhibiting Intestinal Cholesterol Absorption
Source: Nutrients. 2023 Dec 9;15(24):5056. doi: 10.3390/nu15245056 (PMC10746001; doi:10.3390/nu15245056)
Supplement: Supplementary file 1 [file nutrients-15-05056-s001.zip › Table S3.pdf]

Table S3. Cecal microbiota: Indicator Genera. Indicator taxa were identified as those whose abundance differ significantly between two groups or more. Relative abundances are presented as the mean ± SD. and p values were adjusted for multiple testing. n=15/16 per group.

	**% of relative abundance**			**Adjusted p. values**			**Total cholesterol level**	
**Genus**	**ND**	**HFD**	**HFD-T070 5%**	**ND vs HFD**	**ND vs T070 5%**	**HFD vs HFD T070 5%**	**Pearson**	**Adj p. values**
Alloscardovia	0.02±0.01	0.00±0.00	0.00±0.00	p =0.00	p =0.00	p =0.46	-0.63	p =0.00
Bifidobacterium	0.30±0.17	0.09±0.05	0.02±0.03	p =0.01	p =0.00	p =0.00	-0.49	p =0.00
uncl. *Bifidobacteriaceae*	0.04±0.02	0.02±0.02	0.00±0.01	p =0.08	p =0.00	p =0.03	-0.39	p =0.02
Olsenella	0.05±0.03	0.01±0.02	0.00±0.00	p =0.05	p =0.00	p =0.07	-0.45	p =0.01
uncl. *Eggerthellaceae*	0.00±0.01	0.01±0.01	0.05±0.03	p =0.86	p =0.00	p =0.00	0.03	p =0.91
Adlercreutzia	0.02±0.02	0.01±0.01	0.04±0.03	p =0.49	p =0.05	p =0.00	-0.14	p =0.57
Raoultibacter	0.00±0.00	0.00±0.00	0.01±0.01	p =0.49	p =0.03	p =0.01	-0.07	p =0.79
uncl. *Coriobacteriia*	0.03±0.02	0.05±0.03	0.08±0.05	p =0.25	p =0.00	p =0.14	0.11	p =0.60
uncl. *Muribaculaceae*	3.23±0.55	2.04±0.72	3.11±0.67	p =0.01	p =0.82	p =0.00	-0.59	p =0.00
Paramuribaculum	0.56±0.46	1.16±0.69	0.51±0.28	p =0.07	p =0.83	p =0.01	0.38	p =0.03
Duncaniella	6.67±2.75	4.22±2.03	6.11±2.35	p =0.07	p =0.87	p =0.03	-0.45	p =0.01
Muribaculum	0.18±0.09	0.23±0.18	0.65±0.31	p =0.88	p =0.00	p =0.00	0.04	p =0.88
uncl. *Prevotellaceae*	0.88±0.30	0.88±0.35	1.62±0.40	p =0.88	p =0.00	p =0.00	-0.09	p =0.70
Paraprevotella	4.61±2.30	4.84±3.34	9.18±3.40	p =0.86	p =0.00	p =0.01	-0.13	p =0.57
uncl. *Bacteroidales*	1.60±0.37	1.49±0.44	2.47±0.55	p =0.41	p =0.00	p =0.00	-0.12	p =0.59
Helicobacter	0.16±0.14	0.47±0.43	0.75±0.70	p =0.17	p =0.00	p =0.41	0.19	p =0.34
uncl. *Helicobacteraceae*	0.01±0.01	0.05±0.04	0.08±0.06	p =0.08	p =0.00	p =0.19	0.25	p =0.18
Mucispirillum	0.26±0.15	0.58±0.33	0.90±0.53	p =0.06	p =0.00	p =0.12	0.23	p =0.22
Limosilactobacillus	0.11±0.15	0.03±0.09	0.00±0.01	p =0.07	p =0.00	p =0.90	-0.28	p =0.13
Lactococcus	0.05±0.04	0.11±0.06	0.05±0.03	p =0.08	p =0.89	p =0.03	0.42	p =0.01
uncl. *Lachnospiraceae*	23.60±3.68	14.80±1.88	13.12±2.95	p =0.00	p =0.00	p =0.46	-0.62	p =0.00
Anaerostipes	0.01±0.01	0.00±0.00	0.00±0.01	p =0.03	p =0.01	p =1.00	-0.48	p =0.00
Kineothrix	0.17±0.29	0.07±0.07	0.00±0.00	p =0.49	p =0.01	p =0.00	-0.20	p =0.33
Schaedlerella	0.21±0.16	0.11±0.10	0.56±0.35	p =0.21	p =0.00	p =0.00	-0.14	p =0.57
Anaerotignum	0.11±0.05	0.15±0.06	0.19±0.07	p =0.22	p =0.00	p =0.20	0.27	p =0.14
uncl. *Ruminococcaceae*	7.22±1.50	9.51±2.55	5.23±0.53	p =0.21	p =0.00	p =0.00	0.30	p =0.10
Flintibacter	1.24±0.39	1.97±0.57	1.41±0.23	p =0.02	p =0.35	p =0.02	0.46	p =0.01
Clostridium_IV	0.07±0.12	0.00±0.00	0.00±0.00	p =0.15	p =0.02	p =0.46	-0.35	p =0.05

Neglecta	0.11±0.06	0.07±0.05	0.34±0.11	p =0.18	p =0.00	p =0.00	-0.13	p =0.57
Lawsonibacter	0.45±0.25	0.59±0.22	1.16±0.36	p =0.31	p =0.00	p =0.00	0.03	p =0.91
Ruthenibacterium	0.12±0.08	0.16±0.12	0.31±0.11	p =0.50	p =0.00	p =0.00	0.02	p =0.93
Paludicola	0.10±0.09	0.11±0.06	0.22±0.08	p =0.76	p =0.00	p =0.00	-0.05	p =0.87
Dysosmobacter	0.53±0.31	0.51±0.34	0.19±0.17	p =0.84	p =0.01	p =0.02	-0.05	p =0.86
Allobaculum	3.79±2.59	2.06±2.46	0.31±0.31	p =0.20	p =0.00	p =0.00	-0.29	p =0.12
uncl. *Alphaproteobacteria*	0.41±0.23	0.59±0.39	1.00±0.61	p =0.49	p =0.01	p =0.14	0.08	p =0.73
uncl. *Desulfovibrionaceae*	0.73±0.30	0.50±0.25	0.77±0.22	p =0.18	p =0.46	p =0.02	-0.39	p =0.02
Mailhella	8.85±2.15	13.31±2.46	12.30±1.61	p =0.01	p =0.00	p =0.64	0.57	p =0.00
uncl. *Bacteria*	3.99±1.11	4.55±1.20	6.83±1.99	p =0.50	p =0.00	p =0.00	0.13	p =0.57
uncl. *Opitutae*	0.00±0.00	0.00±0.00	0.00±0.01		p =0.03	p =0.04	0.01	p =0.95
