# Supplementary material for: Totum-070, a Polyphenol-Rich Plant Extract, Prevents Hypercholesterolemia in High-Fat Diet-Fed Hamsters by Inhibiting Intestinal Cholesterol Absorption
Source: Nutrients. 2023 Dec 9;15(24):5056. doi: 10.3390/nu15245056 (PMC10746001; doi:10.3390/nu15245056)
Supplement: Supplementary file 1 [file nutrients-15-05056-s001.zip › Table S4.pdf]

Table S4. List of hamster primers used for qPCR.

Gene	Primers
Abcb1	Forward: TTCGCTATGGCCGAGAAGAC
	Reverse: TCTCACCGACCAGAGTGTCA
Abcb11	Forward: ATCTGGGAAGAGCACAGCAC
	Reverse: CCGGATGTTGAGAGAGCGAA
Abcg5	Forward: CGTGGCAGACCGAATGATTG
	Reverse: GTCCTGTGGTTGGCTCATCA
Abcg8	Forward: GCAACCCCGCTGACTTCTAT
	Reverse: TCATCGAAGCCTCGCACTTT
Acaca	Forward: TTAACAGCGGCAGAGTCAGG
	Reverse: GAAGCTCAGCCTGTTGGACT
Acox1	Forward: TGGCTGTTAAGAAGAGTGCCA
	Reverse: GTAATTGAGGCCCACAGGCT
Adgre1	Forward: GCTTCCAACCAGAGCCAGAA
	Reverse: CCTGCTTGGCACTGCTGTAT
Angptl3	Forward: ACGGTTTTGGAAGATTTGCGG
	Reverse: TGCTTGCTGTCTTTGCAGTC
Apob	Forward: ATTCTGCCAGCGCAACCTAT
	Reverse: GACAAAGTCCTGCTTTGCCG
Apoc3	Forward: TAATGAGGTAGAGGGGTCCTTG
	Reverse: GTAGCTTTTCAGGGAGGTGA
Ccl2	Forward: AACGCTTCTGTGCCTACTGC
	Reverse: TCTTGTAGCTCTCCAGCCTCT
Cidec	Forward: TACGACTTGCACTGCTACCG
	Reverse: TCAGTGGCATCCAGGAACTG
Cldn1	Forward: TGTGGATGTCCTGCGTTTCG
	Reverse: TGCGATCAGTCCCAGAAGGA
Cldn2	Forward: ACGGGCTCCGAAGAGACTTA
	Reverse: ATGCTGGCACCAACGTAAGA
Cpt1a	Forward: TTATCGTGGTGGTGGGTGTG
	Reverse: ATGCCACTCACGACGTTCTT
Cyp7a1	Forward: GGAGCATTACAGAGTGCTGGT
	Reverse: AGGATGCACTGGAAAGCCTC
Cyp27a1	Forward: AGGAGGGCAAGTACCCCATA
	Reverse: TGATGCCCTTGCGTGAGAAA
Cyp8b1	Forward: AGTTCCGCAGGTTTGACCTC
	Reverse: ATGCCGTCTTTCTGCAGGTT
Fabp6	Forward: GGCAACCTTCCCCAACTATCA
	Reverse: AAGCCAGCCTCTTGCTTACA
Fasn	Forward: CACAGTGCTCCGAGGTCATT
	Reverse: CACCAGGTGCAATCCCTTCT
Gbpbar1	Forward: AGAAAAGCCCAGCTAACAAGC
	Reverse: TGTTGGGTGTCATCATGGGTC
Hmgcr	Forward: GCAGATACTGGAGAGTGCCG
	Reverse: GCTGCCGGCGAATAGATACA
Il1b	Forward: GCAACTGTTCCTGAACTCGAC
	Reverse: TGGATAGCTCAGGTCAAGGCT

Il6	Forward: CCTGGAGTTTGTGACGAACAAT<br>Reverse: GTTGGGCTAGGCGTGACTATT
Ldlr	Forward: CTCCACTCTATCTCCAGCATTG<br>Reverse: TTTCAGCCACCAAATTCACATC
Lpl	Forward: TTGTGATCCATGGCTGGACG<br>Reverse: ATCCACCACGATGACGTTGG
Mttp	Forward: ATTCAGCTGCAATCTGGACTAA<br>Reverse: ACGAAAGAAGAGTCCACTGTGA
Npc1l1	Forward: ATGGCCACTCACTGTCTTGG<br>Reverse: CGTCGTGGAAAGCCTTCTCT
Nr1h4	Forward: AGGTTAGCCTTCACCACAGC<br>Reverse: CGCCTCCAAATCCAGGTTCT
Ocln	Forward: AGTGGATGGCAAGCGATCAT<br>Reverse: TACCACTGCTGCTGTACCGA
Pcsk9	Forward: TGCTCCAGAGGTCATCACAG<br>Reverse: GTCCCACTCTGTGACATGAAG
Polr2a	Forward: AGGAACACATGCCACCTTTCA<br>Reverse: GAAATGGGAATGTCACACAGCA
Ppara	Forward: GCGCTGAACATTGAGTGTCG<br>Reverse: ACGCCAGCTTTAGCCGAATA
Slc10a1	Forward: CTCAAGACCAAAAGGCCACAG<br>Reverse: TGTCATGGCGAACTTGATGC
Slc10a2	Forward: TCACAAATGGCCCCAAAAAGC<br>Reverse: GGTTCAATGGTCCAGGCACT
Slc51b	Forward: AGAGTTTCGTGCAAAGACTGC<br>Reverse: CCTGCTCCTGGTTGAGATGTT
Slc51a	Forward: CTGGAGCTGCTGAAAACCAAC<br>Reverse: GTCAAGATGATGGTGAGGGCA
Slco1a5	Forward: CATGGACAGAGTGTGATTTTGGT<br>Reverse: TGCTACCCCCAATGCTTTGAA
Srebf1	Forward: TTGAGGATAGCCAGGTGAAAGC<br>Reverse: ACAGTGATGCCAAGGGGTTG
Srebf2	Forward: GAGAGCTGTGAATTTTCCAGTG<br>Reverse: CTACAGATGATATCCGGACCAA
Tgfb1	Forward: ATTCGGGAAGCAGTGCCTGA<br>Reverse: ACGCCAGGTATTGTTGCTGT
Tjp1	Forward: GCTGGATTCCTGAGACCTGT<br>Reverse: CCAGCATCTCGTGGTTCACT
Tnfa	Forward: CAACCCTATCATCGGCTCCA<br>Reverse: GCTCCTCCACTTGGTGGTTT
